# Porcine CD8α^dim/-^NKp46^high^ NK cells are in a highly activated state

**DOI:** 10.1186/1297-9716-44-13

**Published:** 2013-03-01

**Authors:** Kerstin H Mair, Andrea Müllebner, Sabine E Essler, J Catharina Duvigneau, Anne K Storset, Armin Saalmüller, Wilhelm Gerner

**Affiliations:** 1 Institute of Immunology, Department of Pathobiology, University of Veterinary Medicine Vienna, Veterinärplatz 1, 1210 Vienna, Austria; 2 Institute of Medical Biochemistry, Department of Biomedical Sciences, University of Veterinary Medicine Vienna, Veterinärplatz 1, 1210 Vienna, Austria; 3 Department of Food Safety and Infection Biology, Norwegian School of Veterinary Science, P.O. Box 8146, Dep, N-0033 Oslo, Norway

## Abstract

Natural Killer (NK) cells play a crucial role in the early phase of immune responses against various pathogens. In swine so far only little information about this lymphocyte population exists. Phenotypical analyses with newly developed monoclonal antibodies (mAbs) against porcine NKp46 recently revealed that in blood NKp46^-^ and NKp46^+^ cells with NK phenotype exist with comparable cytotoxic properties. In spleen a third NKp46-defined population with NK phenotype was observed that was characterised by a low to negative CD8α and increased NKp46 expression. In the current study it is shown that this NKp46^high^ phenotype was correlated with an increased expression of CD16 and CD27 compared to the CD8α^+^NKp46^-^ and NKp46^+^ NK-cell subsets in spleen and blood. Additionally NKp46^high^ NK cells expressed elevated levels of the chemokine receptor CXCR3 on mRNA level. Functional analyses revealed that splenic NKp46^high^ NK cells produced much higher levels of Interferon-γ and Tumor Necrosis Factor-α upon stimulation with cytokines or phorbol-12-myristate-13-acetate/Ionomycin compared to the other two subsets. Furthermore, cross-linking of NKp46 by NKp46-specific mAbs led to a superior CD107a expression in the NKp46^high^ NK cells, thus indicating a higher cytolytic capacity of this subset. Therefore porcine splenic NKp46^high^ NK cells represent a highly activated subset of NK cells and may play a profound role in the immune surveillance of this organ.

## Introduction

Natural Killer (NK) cells were initially characterised by their spontaneous lytic activity against certain tumor and virus-infected cells [1,2]. Besides their role as cytotoxic cells through the production of perforin and granzymes, NK cells are potent producers of cytokines like Interferon (IFN)-γ and Tumor Necrosis Factor (TNF)-α [3] and thus play important roles in immunomodulation and the defence against viral, parasitic and bacterial pathogens [4]. A considerable number of phenotypically and functionally different NK-cell subsets have been identified up to date [5]. For example, human NK cells can be divided into functionally and also developmentally distinct subsets according to their differing expression of CD56 in combination with CD16 [6,7] and more recently CD11b and CD27 [8]. In the mouse likewise CD27 and CD11b (Mac-1) are used to dissect NK cells into functionally and developmentally different subsets [9]. Additionally, the chemokine receptor CXCR3 is used in combination with CD27 to distinguish NK-cell subsets in the mouse [10].

For porcine NK cells a perforin^+^CD2^+^CD3^-^CD4^-^CD5^-^CD6^-^CD8α^+^CD8ß^-^CD11b^+^CD16^+^ phenotype has been described and it was shown that these lymphocytes can perform immediate cytotoxicity against NK-susceptible targets [11-13]. Moreover, in parasitic as well as in viral infections increases in NK cell number and activity have been reported [14,15], but also inhibitory effects on NK-cell mediated cytotoxicity and cytokine production by viral infections are described [16-19]. Despite these hints on important functions of porcine NK cells in vivo, so far no investigations on the existence of functionally differing NK-cell subsets have been reported. Nevertheless, a recent study from our group with newly developed monoclonal antibodies (mAbs) against the activating receptor NKp46 enabled a more comprehensive insight into the phenotype of porcine NK cells and putative subsets [20].

NKp46 (CD335, NCR1) is a member of the natural cytotoxicity receptor (NCR) family, which is involved in the control of tumors and viral infections [21-26]. Moreover, it has been used as a marker for NK cell identification in different species like humans [27,28], monkeys [29,30], rodents [30-32], cattle [33] and more recently in sheep [34] and horses [35]. In contrast, NKp46 in the pig was shown to divide porcine CD3^-^CD8α^+^ NK cells into NKp46^-^ and NKp46^+^ subsets in blood and all organs tested [20]. CD3^-^CD8α^+^NKp46^-^ NK cells show phenotypic and functional properties of NK cells although they produce reduced levels of IFN-γ compared to the NKp46^+^ subset after in vitro stimulation. Additionally, a third NK cell population with elevated NKp46 expression levels was identified in high frequencies in spleen and liver, pointing towards a special role of NK cells with this phenotype.

Therefore, in this study we focused on functional and phenotypical properties of CD8α^dim/-^NKp46^high^ NK-cells in the spleen. We observed that these cells differ in their expression of various NK-cell associated markers including CD16, the TNF-receptor family member CD27 and the chemokine receptor CXCR3 compared to the CD8α^+^NKp46^-^ and NKp46^+^ NK-cell subsets. Additionally, this NK-cell subset showed an increased cytokine production and cytolytic activity. Thus, our data indicates that CD8α^dim/-^NKp46^high^ NK cells in the pig are in a highly activated state.

## Material and methods

### Isolation of porcine lymphocytes

Blood and spleens were obtained from 6–7 month-old healthy pigs from an abattoir. Animals were subjected to electric high voltage anaesthesia followed by exsanguination. This procedure is in accordance to the Austrian Animal Welfare Slaughter Regulation. Peripheral blood mononuclear cells (PBMC) were isolated using density gradient centrifugation (Lymphocyte Separation Medium, density: 1.077 g/mL, PAA, Pasching, Austria) as described previously [36]. Dissected spleen was cut into small pieces and mechanically dissociated by forcing through a sieve. After a washing step in phosphate buffered saline (PBS, PAA) cells were applied to density gradient centrifugation to isolate mononuclear cells. Isolated lymphocytes were finally resuspended in culture medium or PBS containing 10% (v/v) porcine plasma for analysis by flow cytometry (FCM).

### Cell culture

Isolated porcine PBMC and splenocytes were cultivated in RPMI 1640 (PAA) with stable glutamine supplemented with 10% (v/v) heat inactivated foetal calf serum (FCS, PAA), 100 IU/mL penicillin and 0.1 mg/mL streptomycin (PAA). Medium for sorted NK cells was additionally supplemented with 1 mM sodium pyruvate (PAA), non-essential amino acids (PAA) and 50 μM 2-mercaptoethanol (Sigma-Aldrich, Vienna, Austria). Where indicated, cells were additionally cultured in the presence of various cytokines as outlined below.

### Flow cytometry and antibodies

Freshly isolated PBMC or splenocytes were resuspendend in PBS containing 10% (v/v) porcine plasma and labelled for flow cytometric analysis. Cultured cells were resuspended in PBS containing 3% (v/v) FCS for FCM staining. All incubation steps were performed for 20 min on ice. The following primary antibodies were used for cell surface staining: unconjugated or Alexa647-conjugated anti-NKp46 (IgG1, clone VIV-KM1, [20]), anti-CD3 (IgG2b, clone BB23-8E6, Southern Biotech, Birmingham, AL, USA), PerCP-Cy5.5-conjugated anti-CD3 (IgG2a, clone BB23-8E6-8C8, BD Biosciences, San Jose, CA, USA), eFluor450-conjugated anti-CD3 (IgG1, clone PPT3, custom-conjugation by eBioscience, San Jose, CA, USA), unconjugated or FITC-conjugated anti-CD8α (IgG2a, clone 11/295/33), PE-conjugated anti-CD8α (IgG2a, clone 76-2-11, BD Biosciences), anti-CD16 (IgG1, clone G7, Serotec, Raleigh, NC, USA), Biotin-conjugated anti-CD27 (IgG1, clone b30c7, [37]). All non-commercial monoclonal antibodies were produced in-house [38]. Where indicated, these antibodies had been purified and covalently conjugated to fluorochromes or Biotin. Alexa Fluor-647 Protein Labelling Kit (Life Technologies, Carlsbad, CA, USA) was used for conjugation of anti-NKp46 mAbs according to manufacturer’s instructions. FITC conjugation for anti-CD8α mAbs was performed as described elsewhere [39]. Anti-CD27 mAbs were biotinylated using Sulfo-NHS-LC-Biotin (Thermo Scientific, Pierce, Vienna, Austria) following manufacturer’s instructions. Unspecific binding was assessed by appropriate isotype-matched control antibodies. For indirect labelling, anti-mouse anti-IgG1-PE (Southern Biotech) and Streptavidin-Brilliant Violet 605 conjugate (BioLegend, San Jose, CA, USA) were used as second-step reagents. To discriminate between live and dead cells, Fixable Near-IR Dead Cell Stain Kit (Life Technologies) was used according manufacturer’s protocol with 0.05 μL reactive dye per reaction. If unconjugated and conjugated antibodies with the same isotype were used in combination, a sequential staining was performed. Unconjugated primary mAb was used in a first step, followed by isotype-specific dye-conjugated antibodies. After secondary incubation, free binding sites of mouse-isotype specific antibodies were blocked by whole mouse IgG molecules (2 μg per sample, Jackson ImmunoResearch, Suffolk, UK) followed by a further incubation step with fluorochrome-conjugated primary mAbs.

FCM analyses were performed on a FACSCanto II or FACSAria (BD Biosciences). Data of at least 5 × 10^4^ lymphocytes per sample were recorded. Data were analysed with FACSDiva software (Version 6.1.3, BD Biosciences) and FlowJo software (Version 7.6.3., Tree Star, Ashland, OR, USA). Box plots were created by SigmaPlot software (Version 11.0, Systat Software Inc., Erkrath, Germany).

### Intracellular staining of IFN-γ

For intracellular staining of IFN-γ, PBMC and splenocytes were stimulated in 96-well round-bottom plates at 2 × 10^5^ cells per well in a final volume of 200 μL. Cells were either stimulated with 30 IU/mL recombinant human Interleukin (rhIL)-2 (Roche, Vienna, Austria) in combination with 25 ng/mL recombinant porcine Interleukin (rpIL)-12 and 100 ng/mL rpIL-18 (both R&D Systems, Minneapolis, MN, USA) overnight, or left in medium alone as negative control. For IFN-γ labelling, Brefeldin A (GolgiPlug, BD Biosciences) was added to microcultures at a final concentration of 1 μg/mL, 4 h prior to harvest. Cells were labelled with antibodies against CD3, CD8α and NKp46 as stated above. Afterwards cells were fixed and permeabilized as described elsewhere [40] and labelled with anti-IFN-γ-PE (IgG1, clone P2G10, BD Biosciences) as well as corresponding isotype control mAb (mouse-IgG1-PE, clone MOPC-21, BD Biosciences).

### Fluorescence-activated cell sorting (FACS) of NK cells

For sorting of CD3^-^CD8α^+^NKp46^-^ and CD3^-^CD8α^+^NKp46^+^ NK cells of blood as well as CD3^-^CD8α^+^NKp46^-^, CD3^-^CD8α^+^NKp46^+^ and CD3^-^CD8α^dim/-^NKp46^high^ NK cells from spleen, isolated mononuclear cells were labelled with primary antibodies against CD3, CD8α and NKp46 as described above. As secondary antibodies anti-IgG1-PE (Southern Biotech), anti-IgG2a-Alexa647 and anti-IgG2b-Alexa488 (both Life Technologies) were used. PBS containing 5% (v/v) FCS and 2 mM EDTA was used for all washing steps. Sorting was performed on a FACSAria (BD Biosciences). Purity of sorted cell populations was at least 97.5% or higher. Sorted cells were either transferred directly into cell culture or resuspended in TRI Reagent (Sigma-Aldrich) and stored at −80°C for subsequent mRNA analysis.

### CD107a degranulation assay

NK cell receptor mediated degranulation was assessed by measuring the expression of CD107a on the cell surface in combination with four-color flow cytometry to discriminate between the different NK-cell subsets. Degranulation assays were performed according to a protocol for human NK cells [41] and modified as follows. Triggering of NK-receptors was performed by using monoclonal antibodies against NKp46 (IgG1, clone VIV-KM3, [20]), CD16 (IgG1, clone G7, Serotec) or a combination of both. Monoclonal antibodies were coated on 96-well round-bottom wells by incubation overnight at 4°C at a concentration of 3 μg/mL each in PBS in a total volume of 50 μL per well. Isotype-matched irrelevant antibodies served as control (6 μg/mL in 50 μL per well). Plates were washed with PBS for three times before cells were added.

Freshly isolated PBMC and splenocytes were stimulated with rhIL-2 (25 IU/mL) and rpIL-15 (15 ng/mL, Biosource, Nivelles, Belgium) overnight with 2 × 10^5^ cells in a total volume of 200 μL per well, using 96-well round-bottom plates. Since NKp46 was rapidly internalised after receptor triggering, cells were labelled with Alexa647-conjugated anti-NKp46 mAb prior to transfer into antibody-coated plates. The simultaneous use of VIV-KM1 for fluorescence-staining and VIV-KM3 for coating of plates was possible because the two mAbs bind to different sites on NKp46 [20]. After two washing steps to eliminate unbound anti-NKp46-Alexa647 antibodies, cells were used at a concentration of 2 × 10^5^ cells in a total volume of 200 μL per well (mAb-coated 96-well round-bottom plate) in the degranulation assay. Additionally, microcultures were supplemented with FITC-conjugated anti-CD107a mAb (IgG1, clone 4E9/11, Serotec) at a final concentration of 4 μg/mL and the two protein transport inhibitors Brefeldin A (GolgiPlug, final concentration 1 μg/mL) and Monensin (GolgiStop, final concentration 2 μg/mL) (both BD Biosciences). After an incubation of one hour at 37°C, cells were re-labelled with Alexa647-conjugated anti-NKp46 in combination with PE-conjugated anti-CD8α and eFluor450-conjugated anti-CD3 monoclonal antibodies for FCM as described above.

### Analysis of IFN-γ and TNF-α production by ELISA

FACS-sorted NK-cell subsets from blood and spleen were stimulated in 96-well round-bottom plates at 2 × 10^5^ cells in a final volume of 200 μL per well for cytokine production. For IFN-γ and TNF-α production cells were stimulated with a combination of rhIL-2 (30 IU/mL), rpIL-12 (25 ng/mL) and rpIL-18 (100 ng/mL). TNF-α production was also analysed after stimulation with 50 ng/mL phorbol-12-myristate-13-acetate (PMA) and 500 ng/mL Ionomycin (both Sigma-Aldrich). After 24 h, supernatants were collected and tested for cytokine production with commercially available ELISA Kits for IFN-γ (Mabtech, Nacka Strand, Sweden) and TNF-α (R&D Systems) according to manufacturers’ protocols. Optical densities (ODs) were measured at 450/620 nm with an ELISA reader (Tecan, Sunrise, Crailsheim, Germany).

### Analysis of gene expression by quantitative reverse-transcriptase PCR (RT-qPCR)

Total RNA from FACS-sorted NK-cell subsets from blood and spleen was isolated using TRI Reagent (Sigma-Aldrich) according to manufacturer’s protocol. RNA quality control and cDNA synthesis were performed as described elsewhere [42]. Expression of target genes was determined by real-time PCR, using an internal standard as calibrator. The internal standard (IS) was generated by pooling equal aliquots of the cDNA samples investigated in this study. Primers for target genes were designed using either the public domain programmes Primer3 [43] or Primer-BLAST [44] for CXCR3. All primers were synthesised commercially (Eurofins MWG Operon, Ebersberg, Germany). Sequence information of used primers is listed in Table 1. Whenever possible, primers were forced to span over exon junctions in order to increase specificity. For amplification of target genes SYBR® green I (0.5×, Sigma-Aldrich) was used as reporter dye. The qPCR reaction-mixes contained iTaq® DNA polymerase (0.3 U/reaction, Bio-Rad, Hercules, CA, USA), gene specific primers (250 nmol/L each), a final concentration of 200 μmol/L dNTP each and 3 mmol/L MgCl_2_ (for CXCR3 1.5 mmol/L were used) within provided reaction buffer (1×, Bio-Rad). qPCR was performed on a CFX96™ (Bio-Rad), PCR conditions are listed in Additional file 1. Optimisation and validation of the qPCR assays with target gene-specific primers are likewise described in more detail in Additional file 1. Specificity of the generated PCR products using a cDNA pool of samples was further verified by automated sequencing using the pGEM-T Easy vector system (Promega, Madison, WI, USA) and M13 standard sequencing primer (Eurofins MWG Operon). The multiplex qPCR assay for the reference genes (β-Actin, Cyclophilin A and GAPDH) that were used to normalise each target-gene expression was performed as previously described [45]. Each plate contained corresponding randomly assigned RT-minus controls (10% of all samples investigated), the no-template controls (NTC), as well as the IS. All samples were measured in duplicates.

**Table 1:** Primers used in the RT-qPCR assays of sorted porcine NK cells

**Target gene – accession number**	**Target**	**Primer sequences**	**Position on + strand**	**Product length (bp)**	**Product melt.temp (°C)**
		**forward (F) and reverse (R)**			
NM_001123143	**NKp46**	primers as published in [20], rtprimerdb ID: 8346		110	87.0
EU282355.1	**NKp30**	**F:** TCTATTACCAGGGCAAATGTGAAGT	345	209	91.0
		**R:** GTCACTGGGGTCTAGAATCACTCAT	554		
NM_213813.1	**NKG2D**	**F:**ACAGCAGAGAAGACCAGGATTTCTTCA	598	104	82.0
		**R:** GGAACCATCTTCCCACTGCCAGG	702		
XM_003135179.3	**CXCR3**	**F:** CCGACCACAAGCACCAAAGCA	−69	94	90.0
		**R:** TGGCGTTGGCTCATCTCAGGGA	25		

Sequences of primers (5´-3´) for target genes as well as primer positions on (+) strand, length of specific product in base pairs (bp) and product melting temperature in °C are indicated.

Data were analysed using the CFX manager software (Bio-Rad) in the linear regression mode. For the quantification we applied the method described elsewhere [45]. Target gene expression was displayed as 2^-ΔΔCq values representing the fold changes relative to IS.

### Statistical analysis

Data was analysed for statistical significance by SPSS® (SPSS Statistics Version 20.0, IBM Corp., Armonk, NY, USA). Datasets with two groups (PBMC) were analysed using paired two-tailed Student’s *T*-test. For datasets containing more than two groups (spleen) one-way variance analysis with Bonferroni correction for paired sample means was applied. If sample size per group was < 4, no statistical evaluation was performed. Three different levels of significance were defined: *p* < 0.05 (indicated by *), *p* < 0.01 (indicated by **) and *p* < 0.001 (indicated by ***).

## Results

### Splenic NKp46^high^ NK cells vary in their expression of NK-cell associated surface markers

**Figure 1:**
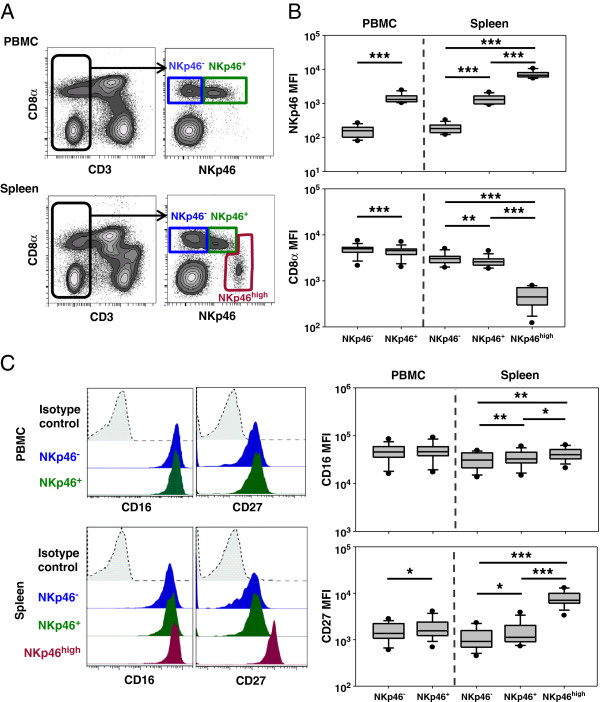
**Varying expression of NKp46, CD8α, CD16 and CD27 on NK-cell subsets in blood and spleen.** (**A**) Following five-colour staining, PBMC and splenocytes were gated on CD3^-^ cells and further subgated according to their NKp46/CD8α expression pattern in NKp46^-^ and NKp46^+^ NK cells in blood (upper graph) and NKp46^-^, NKp46^+^ and NKp46^high^ NK cells in spleen (lower graph). (**B**) 14 healthy 6–7 month old pigs were investigated for NKp46 and CD8α expression levels in the NKp46-defined NK-cell subsets. Box-plots show the mean fluorescence intensity of the two markers. (**C**) NK-cell subsets defined in (**A**) were further analysed for their expression of the surface markers CD16 and CD27. Histograms show the expression of the two markers within the respective NKp46-defined subsets (CD8α^+^NKp46^-^: blue histograms, CD8α^+^NKp46^+^: green histograms, CD8α^dim/-^NKp46^high^: red histograms) in blood (upper graphs) and spleen (lower graphs) according to the corresponding isotype control (grey histrograms with dotted lines). Box-plots show the mean fluorescence intensity of CD16 and CD27 of the NKp46-defined NK-cell subsets in blood and spleen of 14 healthy 6–7 month old pigs. (**B** + **C**) Significant differences between the subsets in blood or spleen are indicated (* = p < 0.05, ** = p < 0.01, *** = p < 0.001).

To expand the knowledge about the phenotype of previously described NKp46-defined NK-cell populations in swine [20], we performed flow cytometric analyses of lymphocytes isolated from spleen and blood. A gating hierarchy was used throughout the experiments to exclude doublets, dead cells as well as CD3^+^ T cells. Remaining CD3^-^ lymphocytes were further analysed for CD8α and NKp46 expression (see Additional file 2). As previously described [20], among CD3^-^ lymphocytes two NK populations could be found in blood, namely NKp46^-^ and NKp46^+^ cells that were both CD8α^+^ (Figure 1A, upper graph). The third NKp46-defined subset that was found in spleen (Figure 1A, lower graph) was characterised by a low to negative CD8α expression and increased expression of the activating receptor NKp46. This applied to all animals analysed, resulting in a mean fluorescence intensity (MFI) for NKp46 that was 4–7 times higher than in the splenic NKp46^+^ subset (7258 ± 1649 to 1362 ± 397 respectively, Figure 1B, upper graph). CD8α^+^NKp46^+^ cells in blood and spleen showed comparable expression levels of the activating receptor (1462 ± 437 and 1362 ± 397 respectively). We then investigated differences in the expression level of CD8α in the respective NK-cell subsets. Splenic NKp46^high^ NK cells showed a significantly reduced level of CD8α expression compared to the other splenic NK-cell subsets in all animals analysed (NKp46^high^: 495 ± 219, NKp46^+^: 2702 ± 647, NKp46^-^: 3098 ± 818, Figure 1B, lower graph). Of note, NKp46^-^ and NKp46^+^ NK cells in blood as well as spleen also showed a differential expression of CD8α, although it was not as obvious as for the NKp46^high^ subset. Within each location NKp46^-^ NK cells showed the highest expression of CD8α, thus indicating a correlation between an increase of NKp46 and a decrease of CD8α expression on porcine NK-cell subsets.

To get more insight into the phenotype of the NKp46-defined NK cells we further analysed the expression of CD16 and the TNF-receptor family member CD27. The latter is an important marker to distinguish between NK-cell subsets in mouse and human [8-10,46] and the porcine orthologue of CD27 was recently identified [37]. No marked difference in CD16 or CD27 expression between blood NKp46^-^ and NKp46^+^ NK-cell subsets could be observed, although NKp46^+^ cells showed a slightly increased CD27 expression (Figure 1C). A clear difference between the three splenic NKp46-defined NK-cell subsets could be observed for CD16 (NKp46^high^: 42 588 ± 12 000, NKp46^+^: 35 521 ± 12 419, NKp46^-^: 31 285 ± 11 267, Figure 1C) and was even more obvious for CD27 expression (NKp46^high^: 7978 ± 2866, NKp46^+^: 1562 ± 909, NKp46^-^: 1149 ± 604, Figure 1C). NKp46^-^ NK cells showed the lowest and NKp46^high^ NK cells the highest expression of both markers, thus indicating a positive correlation between NKp46, CD16 and CD27 expression levels.

### Splenic NKp46^high^ NK cells produce the highest levels of cytokines

**Figure 2:**
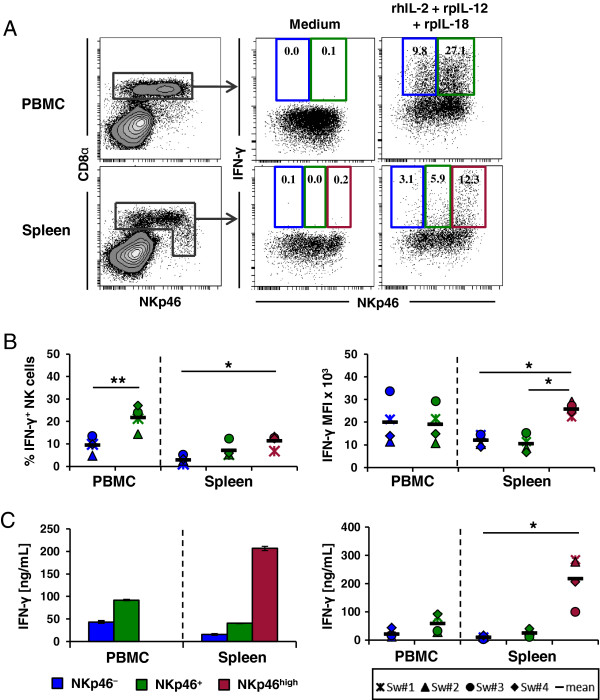
**Splenic NKp46**^**high **^**NK cells produced the highest levels of IFN-γ.** (**A** + **B**) Intracellular cytokine staining for IFN-γ production in NKp46-defined NK cells within PBMC (upper graphs) and splenocytes (lower graphs) by four-colour FCM after 24 h in vitro stimulation with rhIL-2, rpIL-12 and rpIL-18 or medium. CD3^-^ lymphocytes were gated (not shown) and NKp46/CD8α defined total NK cells were further subgated for IFN-γ production. IFN-γ producing NK cells were gated according to NKp46 expression levels (CD8α^+^NKp46^-^: blue, CD8α^+^NKp46^+^: green, CD8α^dim/-^NKp46^high^: red). (**A**) Numbers indicate the percentage of IFN-γ^+^ NK cells within respective gates. Results are representative for experiments with four different animals. (**B**) Percentage of IFN-γ^+^ cells (left graph) and mean fluorescence intensity of IFN-γ^+^ cells (right graph) within the different NK-cell subsets in blood and spleen of four animals are shown. (**C**) Supernatants of cytokine stimulated FACS-sorted CD3^-^CD8α^+^NKp46^-^ and CD3^-^CD8α^+^NKp46^+^ NK cells from blood and CD3^-^CD8α^+^NKp46^-^, CD3^-^CD8α^+^NKp46^+^ and CD3^-^CD8α^dim/-^NKp46^high^ NK cells from spleen were tested for IFN-γ production in ELISA following 24 h in vitro stimulation with rhIL-2, rpIL-12 and rpIL-18. Data on the left are from one representative animal and displayed as the mean of duplicates ± SD. IFN-γ production in experiments with four animals analysed is shown on the right. Mean values are represented by a black bar. (**B** + **C**) Significant differences between the subsets in blood or spleen are indicated (* = p < 0.05, ** = p < 0.01).

In human and mice higher CD27 expression on NK cells is associated with an increased cytokine production [9,10,46,47]. If the same holds true for porcine NK cells, splenic NKp46^high^CD27^high^ NK cells should be the most prominent cytokine producers compared to the other subsets. We therefore compared IFN-γ and TNF-α production between the different NK-cell populations in blood and spleen of several individuals (*n* = 4). Intracellular staining for IFN-γ in total PBMC and splenocytes was performed after in vitro stimulation with a combination of rhIL-2, rpIL-12 and rpIL-18 overnight. Cells cultured in medium alone served as negative control and did not show any IFN-γ production. The percentage of IFN-γ^+^ NK cells was significantly higher in the blood NKp46^+^ NK-cell subset compared to blood NKp46^-^ cells after cytokine stimulation (21.7% ± 4.7 versus 9.7% ± 3.1, respectively, Figure 2A and B). Nevertheless, the produced amount of IFN-γ per cell, investigated by the MFI, was similar for both blood NK-cell subsets (19 072 ± 7009 for NKp46^+^ and 20 006 ± 8634 for NKp46^-^, Figure 2B, right graph). Splenic NKp46^high^ NK cells clearly showed the highest frequency of IFN-γ^+^ cells compared to the other two splenic subsets whereas NKp46^-^ cells had the lowest frequency (NKp46^high^: 11.2% ± 3.6%, NKp46^+^: 7.2% ± 3, NKp46^-^: 2.9% ± 1.5, Figure 2A and B). Additionally, the amount of IFN-γ produced per cell was considerably higher in the NKp46^high^ subset (NKp46^high^: 25 790 ± 2615, NKp46^+^: 10 432 ± 3211, NKp46^-^: 12 008 ± 2396, Figure 2B, right graph). Yet, stimulated splenic NK-cell subsets showed an overall lower frequency of IFN-γ producing cells compared to blood.

Results of intracellular cytokine staining were confirmed by ELISA (Figure 2C). FACS-sorted CD3^-^CD8α^+^NKp46^-^ and CD3^-^CD8α^+^NKp46^+^ NK cells from blood and CD3^-^CD8α^+^NKp46^-^, CD3^-^CD8α^+^NKp46^+^ and CD3^-^CD8α^dim/-^NKp46^high^ NK cells from spleen were stimulated with rhIL-2, rpIL-12 and rpIL-18 overnight and supernatants were tested for IFN-γ production. Blood NKp46^+^ NK cells produced higher levels of IFN-γ (2 to 3-fold) compared to the blood NKp46^-^ subset (58 ± 28 ng/mL versus 21 ± 13 ng/mL, respectively, Figure 2C), which is consistent with the data obtained by flow cytometry. In spleen differences between the NK-cell subsets were much more pronounced as NKp46^high^ NK cells showed a 5 to 20-fold higher IFN-γ production compared to the NKp46^+^ (217 ± 74 ng/mL to 25 ± 12 ng/mL, Figure 2C), and 14 to 50-fold higher production compared to the NKp46^-^ NK cells (217 ± 74 ng/mL to 9 ± 5 ng/mL).

**Figure 3:**
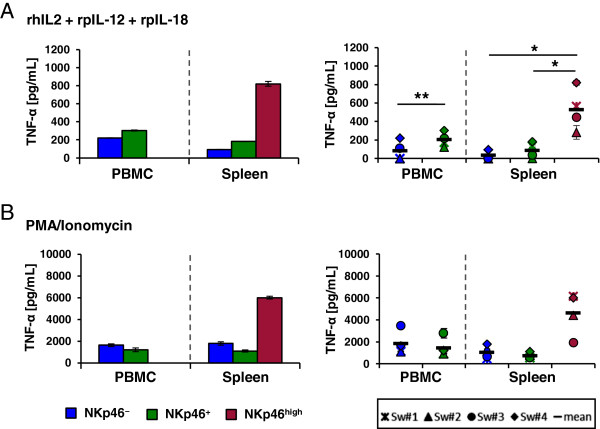
**Splenic NKp46**^**high **^**NK cells produced the highest levels of TNF-α.** Analysis of TNF-α production of NKp46-defined NK-cell subsets (CD8α^+^NKp46^-^: blue, CD8α^+^NKp46^+^: green, CD8α^dim/-^NKp46^high^: red) isolated from blood and spleen. FACS-sorted CD3^-^CD8α^+^NKp46^-^ and CD3^-^CD8α^+^NKp46^+^ NK cells from blood and CD3^-^CD8α^+^NKp46^-^, CD3^-^CD8α^+^NKp46^+^ and CD3^-^CD8α^dim/-^NKp46^high^ NK cells from spleen were stimulated with rhIL-2, rpIL-12 and rpIL-18 **(A)** or PMA and Ionomycin **(B)** for 24 h. Supernatants were tested for TNF-α production in ELISA. Bar graphs on the left show data from one representative animal and are displayed as the mean of duplicates ± SD. TNF-α production of four animals analysed are shown on the right. Mean values are represented by a black bar. Significant differences between the subsets in blood or spleen are indicated (* = *p* < 0.05, ** = p < 0.01).

In addition to IFN-γ, TNF-α production of the different FACS-sorted NK-cell subsets was measured by ELISA after cytokine or PMA/Ionomycin stimulation overnight (Figure 3). After stimulation with rhIL-2, rpIL-12 and rpIL-18, splenic NKp46^high^ NK cells likewise showed the highest levels of TNF-α. Thus TNF-α production was 4 to 12-fold higher in the NKp46^high^ NK-cell subset compared to splenic NKp46^+^ NK cells (530 ± 196 pg/mL to 96 ± 64 pg/mL, Figure 3A) and 9 to 15-fold compared to NKp46^-^ NK cells (530 ± 196 pg/mL to 52 ± 26 pg/mL, Figure 3A). Blood NKp46^+^ NK cells showed a 1.5 to 6-fold higher TNF-α production compared to the blood NKp46^-^ NK-cell subset (204 ± 66 pg/mL to 97 ± 78 pg/mL, Figure 3A). No obvious differences could be observed for TNF-α production between blood or spleen NKp46^-^ and NKp46^+^ NK-cell subsets after PMA/Ionomycin stimulation (Figure 3B) whereas splenic NKp46^high^ NK cells again showed an increased TNF-α production compared to splenic NKp46^+^ (3 to 11-fold, 4629 ± 1704 pg/mL to 748 ± 224 pg/mL, Figure 3B) and splenic NKp46^-^ NK cells (3 to 14-fold, 4629 ± 1704 pg/mL to 1060 ± 540 pg/mL).

Data from both, IFN-γ as well as TNF-α production indicated that splenic NKp46^high^ NK cells, that also displayed an increased CD27 expression, are the most potent cytokine producing NK cell subset.

### Splenic NKp46^high^ NK cells show a superior cytolytic activity after triggering of activating receptors

**Figure 4:**
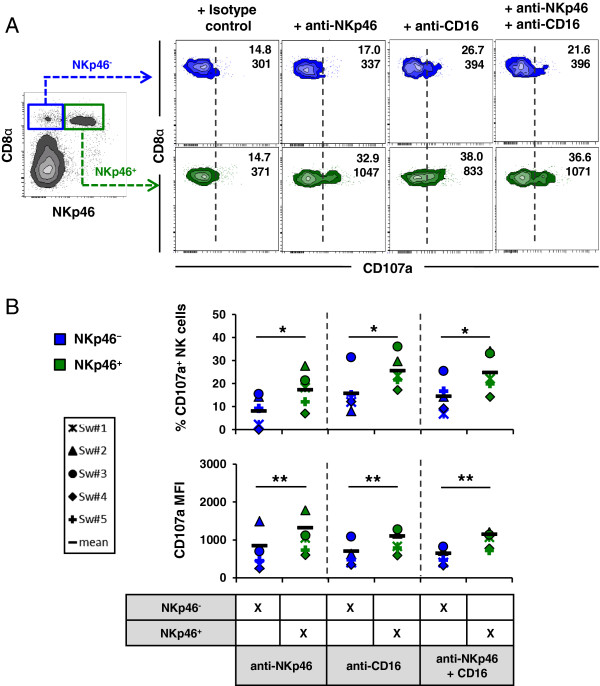
**Analysis of degranulation capacity in NKp46-defined NK-cell subsets in blood.** The cytolytic capacity of NKp46-defined NK-cell subsets (CD8α^+^NKp46^-^: blue, CD8α^+^NKp46^+^: green) isolated from blood was analysed after receptor triggering. Cells were stimulated with rhIL-2 and rpIL-15 overnight. Triggering of NK-receptors was performed by using monoclonal antibodies against NKp46, CD16 or a combination of both. Irrelevant isotype-matched antibody served as negative control. NK-cell receptor mediated degranulation was assessed by measuring the expression of CD107a on the cell surface by four-colour flow cytometry after one hour incubation. CD107a expression was measured on CD3^-^ lymphocytes (not shown), followed by gating on the respective NKp46-defined NK subsets. (**A**) Numbers indicate the percentage of CD107a^+^ cells and the mean fluorescence intensity of CD107a within respective NKp46-gates. Results are representative of experiments with five different animals. (**B**) CD107a expression analyses of five animals analysed. The proportion of CD107a^+^ cells within the different NKp46-defined subsets is shown in the upper graphs. Percentage of CD107a^+^ NK cells was calculated by subtracting spontaneous degranulation observed in cultures stimulated with isotype-control antibodies from the frequency of CD107a^+^ cells in cultures stimulated with NKp46 and/or CD16 mAbs. The lower graphs show the mean fluorescence intensity of CD107a within the respective subsets. Mean values are represented by a black bar. Significant differences between the subsets are indicated (* = p < 0.05, ** = p < 0.01).

**Figure 5:**
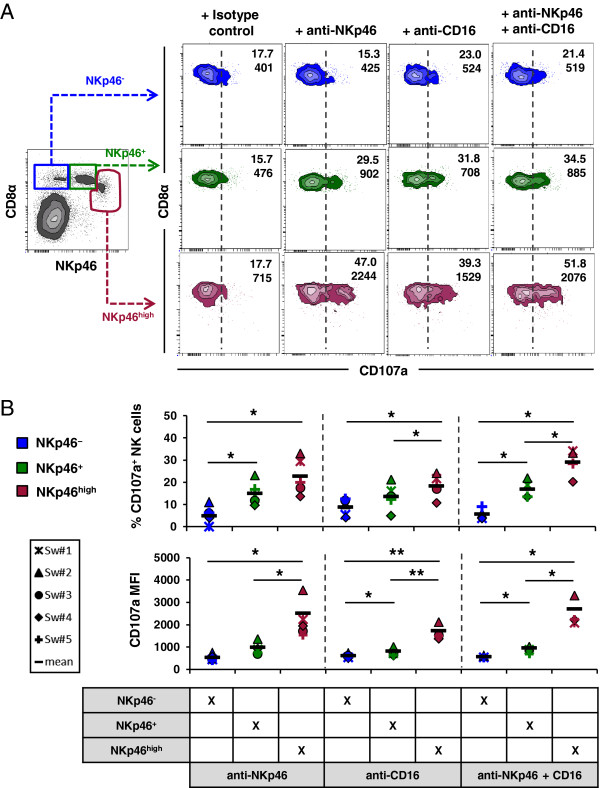
**NKp46**^**high **^**NK cells in spleen showed the highest cytolytic capacity.** The cytolytic capacity of NKp46-defined NK-cell subsets (CD8α^+^NKp46^-^: blue, CD8α^+^NKp46^+^: green, CD8α^dim/-^NKp46^high^: red) isolated from spleen was analysed after receptor-mediated degranulation. Cells were stimulated and gated for flow cytometric analysis as outlined in Figure 4. (**A**) Numbers indicate the percentage of CD107a^+^ cells and the mean fluorescence intensity of CD107a within respective NKp46-gates. Results are representative of experiments with five different animals. (**B**) CD107a expression analyses of five animals analysed. The proportion of CD107a^+^ cells within the different NKp46-defined subsets is shown in the upper graphs. Percentage of CD107a^+^ NK cells was calculated by subtracting spontaneous degranulation observed in cultures stimulated with isotype-control antibodies from the frequency of CD107a^+^ cells in cultures stimulated with NKp46 and/or CD16 mAbs. The lower graphs show the mean fluorescence intensity of CD107a within the respective subsets. Mean values are represented by a black bar. Significant differences between the subsets are indicated (* = p < 0.05, ** = p < 0.01).

It was already shown that blood NKp46^-^ and NKp46^+^ NK-cell subsets show comparable cytolytic activity against xenogeneic and allogeneic target cells in a NKp46-independent manner [20]. To investigate whether the NKp46^high^ phenotype was also correlated with an increased cytolytic activity we performed CD107a degranulation assays in combination with multi-colour flow cytometry. We used monoclonal antibodies against NKp46 or CD16 to mimic receptor-specific ligands to look at a possible correlation between receptor density and cytolytic activity of the different NK-cell subsets in blood (Figure 4) and spleen (Figure 5). Background degranulation was determined by using irrelevant-isotype matched control antibodies.

Blood NKp46^+^ NK cells showed a clear cytolytic activity after triggering with anti-NKp46 mAbs indicated by the induction of CD107a (Figure 4). As expected, NKp46^-^ NK cells showed no obvious increase in CD107a expression after anti-NKp46 stimulation. Although blood NKp46^-^ as well as NKp46^+^ NK cells got activated by triggering of the Fc receptor CD16, this stimulation led to a higher cytolytic activation in the NKp46^+^ NK-cell subset (25.6% ± 6.6 compared to 15.7% ± 8.1 in the NKp46^-^ NK cells, Figure 4B). Co-crosslinking of both receptors also led to a higher CD107a expression in the blood NKp46^+^ NK-cell subset. Interestingly, a comparison of data for the three different stimulations of the blood NKp46^+^ NK-cell subset revealed no co-stimulatory effect after co-crosslinking of NKp46 and CD16 (Figure 4A+B).

Similar results were obtained for spleen NKp46^-^ and NKp46^+^ NK-cell subsets (Figure 5). Splenic NKp46^+^ NK cells showed a higher cytolytic activity after triggering with anti-NKp46 and/or anti-CD16 mAbs compared to NKp46^-^ NK cells (15% ± 4.7 to 5.4% ± 4.7 for NKp46 and 13.6% ± 5.4 to 8.7% ± 3.4 for CD16, Figure 5A+B). No obvious difference in the proportion of CD107a^+^ cells as well as CD107a MFI could be observed in the splenic NKp46^+^ NK-cell subset when comparing the different mAb-stimulations for all animals analysed (Figure 5B, *n* = 5). However, NKp46^high^ NK cells showed a strongly increased frequency of CD107a^+^ cells (22.7% ± 7.3) as well as increased CD107a expression level per cell (MFI of 2501 ± 726) after receptor triggering of NKp46. Likewise after triggering of CD16, splenic NKp46^high^ NK cells showed an increased cytolytic activity compared to the other two NK-cell subsets (18.4% ± 4.6 and a MFI of 1725 ± 267, Figure 5A+B). A slight increase of both read-outs after co-receptor triggering with CD16 and NKp46 could be observed for this NK-cell subset compared to the NKp46^+^ cells where no obvious additive effect on degranulation could be found.

The comparison of cytotoxic capability by degranulation assays demonstrated that blood NKp46^+^ NK cells showed an overall higher proportion of CD107a^+^ cells than splenic NKp46^+^ NK cells, regardless which receptor had been activated (mean of all stimulated fractions, blood NKp46^+^ CD107a^+^: 22.5% versus spleen NKp46^+^CD107a^+^: 15.2%). However, CD107a expression levels per cell, analysed by MFI, did not differ strongly (mean of all stimulated fractions, blood MFI NKp46^+^CD107a^+^: 1184 versus spleen MFI NKp46^+^CD107a^+^: 916). Instead, again regardless which receptor had been activated, the blood NKp46^+^ NK-cell subset showed more similar frequencies of CD107a^+^ cells to the spleen NKp46^high^ NK-cell subset (mean of all stimulated fractions, blood NKp46^+^CD107a^+^: 22.5% versus spleen NKp46^high^CD107a^+^: 23.4%).

### Splenic NKp46^high^ NK cells differ in their expression of the activating receptor NKp30 and the chemokine receptor CXCR3

**Figure 6:**
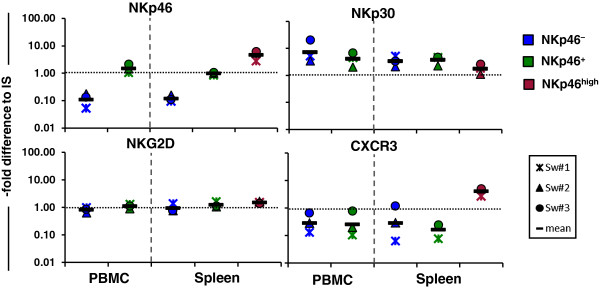
**Varying mRNA expression of NK-associated markers on NKp46-defined NK-cell subsets in blood and spleen.** FACS-sorted CD3^-^CD8α^+^NKp46^-^ and CD3^-^CD8α^+^NKp46^+^ NK cells derived from blood and CD3^-^CD8α^+^NKp46^-^, CD3^-^CD8α^+^NKp46^+^ and CD3^-^CD8α^dim/-^NKp46^high^ NK cells from spleen were analysed for their mRNA expression of various NK-markers by quantitative RT-PCR. Messenger-RNA expression of the NK receptors NKp46, NKp30 and NKG2D as well as the chemokine receptor CXCR3 were analysed in the different NK-cell subsets (CD8α^+^NKp46^-^: blue symbols, CD8α^+^NKp46^+^: green symbols, CD8α^dim/-^NKp46^high^: red symbols). The 2^-ΔΔCt values for each individual animal (*n* = 3) are shown as the fold differences relative to an internal standard (IS = 1, black dotted line). Geometric mean values of the three animals are represented by a black bar.

So far, our functional data indicated that splenic NKp46^high^ NK cells are in an elevated stage of activation. Therefore we further analysed the expression of other NK-associated markers like the NK-receptors NKp30 and NKG2D in this NK-cell subset. Moreover, expression of the chemokine receptor CXCR3 was investigated since the expression of CXCR3 in combination with CD27 can be used for the identification of NK-cell subsets with different functional properties in the mouse [10]. Therefore, FACS-sorted NKp46-defined NK-cell subsets of blood and spleen were analysed for expression of these markers by quantitative RT-PCR (Figure 6). Additionally, NKp46 mRNA levels in the different NK-cell subsets derived from blood and spleen were analysed. Results confirmed data obtained from protein expression, showing the same differential expression of NKp46 mRNA in the respective subsets (Figure 6). Interestingly, despite high NKp46 expression, for the NCR-family member NKp30 a reduced expression was found for splenic NKp46^high^ NK cells compared to the other two splenic subsets. Blood NKp46^-^ NK cells seemed to express slightly higher levels of NKp30 as blood NKp46^+^ NK cells. NKG2D mRNA levels were very homogeneous among the different NKp46-defined subsets. Also, this receptor showed the lowest variation in expression levels between different individuals. The most prominent difference in RNA expression was observed for the chemokine receptor CXCR3. Splenic NKp46^high^ NK cells showed an overall higher expression of this receptor compared to NKp46^-^ and NKp46^+^ NK-cell subsets from both spleen and blood. For NKp46^-^ and NKp46^+^ NK cells from blood and spleen similar expression levels of CXCR3 were observed.

## Discussion

Recently, the phenotype of porcine NK cells was revisited in a study from our group using newly developed mAbs against porcine NKp46. Interestingly, CD3^-^CD8α^+^ NK cells in the blood were shown to be either NKp46^+^ or NKp46^-^[20]. Additionally, a third NK-cell subset with elevated NKp46 expression was found in high frequencies in spleen and liver that was associated with a CD8α^dim/-^ phenotype. In the current study we aimed to investigate this splenic NKp46^high^ NK-cell subset in more detail and elucidate possible functional as well as phenotypical differences to the NKp46^-^ and NKp46^+^ NK-cell subsets in the pig.

Splenic NKp46^high^ NK cells showed highly elevated expression of this activating receptor compared to the other two NKp46-defined subsets in spleen as well as in blood in all animals analysed, which could be demonstrated on protein as well as on mRNA level. Additionally, splenic NKp46^high^ NK cells displayed a strongly decreased expression of CD8α, a receptor that was so far described to be expressed by all NK cells in the pig [12,13]. Of note, also the NKp46^-^ and NKp46^+^ NK cells in blood and spleen showed minor differences in their CD8α expression. We observed a negative correlation, thus an increase of NKp46 expression was accompanied by decreased expression level of CD8α. Furthermore, expression levels of CD16 and the TNF-receptor family member CD27 differed between the three NKp46-defined NK-cell subsets. CD27 expression was clearly enhanced in splenic CD8α^dim/-^NKp46^high^ NK cells and this subset also displayed slightly higher levels of CD16 compared to the other two splenic subsets. In contrast, NKp46^-^ NK cells showed the lowest expression of both receptors, thus indicating a positive correlation in the expression levels of NKp46, CD16 and CD27.

Only few reports highlight on differential expression levels of NKp46 on NK cells in other species, although the existence of NKp46^dull^ and NKp46^bright^ NK cells was already described in the late 1990s on human NK cells [48]. Additionally, it was reported that cells within distinct human NK-cell subsets show different levels of NKp46 expression. Thus, higher CD56 expression [23,49,50] as well as higher CD27 expression [46,47] was associated with higher surface density of NKp46 on human NK cells. Obviously, the latter is akin to the correlation of NKp46 and CD27 we observed in the pig. More recently a phenotype of human NK cells with elevated expression of NKp46 was reported, which show an activated phenotype and seem to play a profound role in hepatitis C virus infection [23]. Likewise to our findings, in that study NKp46^high^ NK cells showed a higher expression of CD27. Furthermore, the authors of this report used the different expression levels of NKp46 to separate NK cells into distinct subsets, similar to the approach of our study.

CD27 is used to distinguish between different NK-cell subsets in mouse and human. In the mouse, CD27 in combination with CD11b is used to differentiate functionally as well as developmentally different NK-cell subsets [9,51]. CD27 divides the mature CD11b^high^ murine NK cells into two distinct subsets. CD27^high^ NK cells show a higher proliferative capacity and additionally an increased cytokine production compared to the CD27^low^ NK-cell subset [9,10]. In regard to cytotoxicity, differing reports exist. It was shown that murine CD27^high^ NK cells show elevated cytolytic activity compared to the CD27^low^ NK-cell subset [9]. However, another report describes the CD27^low/-^CXCR3^-^ NK cells to be the more cytolytic subset in the mouse [10]. In human NK cells, high CD27 expression is also linked to higher cytokine production and the CD27^low/-^ phenotype correlates with an overall higher cytolytic activity [8,46,47]. In contrast, the recent study on human NK cells that used NKp46 to distinguish between different NK-cell subsets showed that NKp46^high^CD27^high^ NK cells have an enhanced cytokine production and cytolytic activity [23]. We likewise observed this bi-functionality in the NKp46^high^CD27^high^ NK-cell subset in spleen, with an elevated IFN-γ and TNF-α production after in vitro stimulation but also higher cytolytic capacity after triggering of the activating receptors CD16 or NKp46 compared to the other two splenic NK-cell subsets. Consistent with this data, it was already suggested that higher density of the activating receptor NKp46 on the NK-cell surface is associated with higher cytolytic function [23,48,50]. Interestingly, we could not observe an obvious synergistic effect after co-crosslinking of both receptors in our study. Nevertheless, similar results were shown for CD16 and NKp46 co-triggering in resting human NK cells [52]. We observed an overall lower cytotoxic activity in the NKp46^-^ subset in blood and spleen when stimulated with anti-CD16 antibody, although the expression level of CD16 was only slightly lower as in the NKp46^+^ NK cells. In our previous study we showed that blood NKp46^-^ porcine NK cells had a cytotoxic capacity comparable to blood NKp46^+^ NK cells in killing assays using xenogeneic and allogeneic cell lines as targets [20]. The lower killing capacity we observed in this study may be caused by the triggering of only a single activation pathway, whereas the killing of target cells is likely to result from the triggering of several activating receptors and/or lack of inhibitory signals.

Although splenic NKp46^high^ NK cells produced higher levels of IFN-γ compared to the other two subsets as shown by ELISA, the overall proportion of IFN-γ producing cells was lower compared to blood NK cells. Therefore, the higher cytokine production seems to result from a superior cytokine production on a single cell level as indicated by the higher MFI for IFN-γ in the splenic NKp46^high^ NK-cell subset. Similar results could be observed for the cytolytic activity determined by CD107a degranulation assays after receptor triggering. Although the proportion of CD107a^+^ cells was not higher in the splenic NKp46^high^ subset compared to blood NKp46^+^ NK cells, NKp46^high^ NK cells showed the highest CD107a expression levels on a per cell basis. Thus, these data may suggest also a higher killing capacity on a single cell level within the splenic NKp46^high^ NK cells. Taken the functional findings together, our data indicate that NKp46^high^ NK cells are in a highly activated state and can readily release high amounts of cytokines or cytolytic granules upon stimulation.

To get further insight into the phenotype of the NKp46-defined NK-cells subsets in the pig we finally performed RT-qPCR analyses of distinct NK-cell associated markers. Expression analyses of other activating receptors, namely NKp30 and NKG2D, showed no marked differences between the NKp46-defined NK cell subsets in blood as well as in spleen. Splenic NKp46^high^ NK cells showed slightly lower levels of the NCR-family member NKp30. NKG2D that is described as important NK-cell receptor involved in target recognition in other species [53] was very uniformly expressed between the different NK-cell subsets in the pig, which is consistent with data in mouse and human where NKG2D shows an overall similar expression pattern between different NK-cell subsets [9,47]. The most prominent difference in expression between the NKp46-defined NK-cell subsets was observed for CXCR3. Splenic NKp46^high^ NK cells showed elevated levels of this chemokine receptor compared to the other two subsets. Likewise, mouse CD27^high^ NK cells showed the highest levels of CXCR3 and recently a further sub-division of murine NK-cells by these two markers has been proposed [10]. The chemokine receptor CXCR3 is associated with the recruitment of NK cells into the lymph node [54] and accumulation in tumors [55]. In the murine spleen, CXCR3 is important for intrasplenic trafficking of NK cells upon inflammatory signals [56] as well as the mobilisation and migration of NK cells from the spleen into the periphery [57]. Thus, one can speculate that the elevated expression of CXCR3 on porcine splenic NKp46^high^ NK cells may indicate that this NK-cell subset is in a “ready-to-go” state for recruitment of NK cells into the periphery or within the spleen.

In conclusion, our data shows that the splenic NKp46^high^ NK cells are in a highly activated state and can be readily activated upon in vitro stimulation. Furthermore NKp46^high^ NK cells are characterised by a distinct receptor expression pattern compared to the NKp46^-^ and NKp46^+^ porcine NK-cell subsets in blood as well as in spleen, including the TNF-receptor family member CD27 that can be used to separate functionally and developmentally different NK-cell subsets in other species.

## Competing interests

The authors declare that they have no competing interests.

## Authors’ contributions

KHM carried out the laboratory work except qPCR analyses and participated in the design of the study. AM performed practical work and data analyses for the qPCR assays. SEE and JCD performed qPCR assay design, validation and carried out qPCR data analyses. AKS contributed to the design of experiments and drafting of the manuscript. AS and WG were responsible for conception and design of the study. KHM and WG wrote the manuscript and analysed the data. All authors read and approved the final manuscript.

## Supplementary Material

Additional file 1 **Optimisation and validation of qPCR assays for NK-associated gene-specific primers.** The suitability of the newly designed primers was verified in separate experiments by performing dilution series of PCR products in 1:10 or cDNA pools in 1:2 steps in quadruplicates. The dilution series, in conjunction with the melt characteristics of the PCR product, were used to optimise the assays regarding the primer concentration, annealing and extension times and the efficiency for the PCR. The optimised PCR conditions including annealing and extension conditions as well as the reaction parameters (slope of the regression analysis corresponding to the efficiency of the qPCR) and the dynamic range for detecting 100% positive of the lowest dilution are indicated in the table. A product was detected in the RT-minus control of some samples, nevertheless these showed at least 5.5 Cqs or more difference to the respective RT-plus sample (ΔCT values are indicated in the table). Calibration curve, melt curve and amplification blot for each target is illustrated. Click here for file

Additional file 2 **Gating hierarchy used for FCM analysis of NKp46-defined NK-cell subsets of porcine PBMC and splenocytes.** (**A**) Lymphocytes were gated according to their light scatter properties. (**B**) To exclude potential doublet cells, a FSC-H/FSC-W gate followed by a SSC-H/SSC-W gate was used. (**C**) For Live/Dead discrimination, Near-IR stain was used. For further analysis only live cells (Near-IR negative) were included. (**D**) To exclude T cells, lymphocytes were further gated on CD3^-^ cells. (**E**) For the identification of different NK subsets CD8α and NKp46 expression was analysed. For PBMC CD3^-^CD8α^+^ cells were divided into NKp46^-^ and NKp46^+^ NK cells. In spleen a third subset could be defined according to its CD8α^dim/-^ and NKp46^high^ phenotype. Click here for file
