# Supplementary material for: Porcine CD8αdim/-NKp46high NK cells are in a highly activated state
Source: Vet Res. 2013 Mar 1;44(1):13. doi: 10.1186/1297-9716-44-13 (PMC3599810; doi:10.1186/1297-9716-44-13)
Supplement: Additional file 1 — Optimisation and validation of qPCR assays for NK-associated gene-specific primers. The suitability of the newly designed primers was verified in separate experiments by performing dilution series of PCR products in 1:10 or cDNA pools in 1:2 steps in quadruplicates. The dilution series, in conjunction with the melt characteristics of the PCR product, were used to optimise the assays regarding the primer concentration, annealing and extension times and the efficiency for the PCR. The optimised PCR conditions including annealing and extension conditions as well as the reaction parameters (slope of the regression analysis corresponding to the efficiency of the qPCR) and the dynamic range for detecting 100% positive of the lowest dilution are indicated in the table. A product was detected in the RT-minus control of some samples, nevertheless these showed at least 5.5 Cqs or more difference to the respective RT-plus sample (ΔCT values are indicated in the table). Calibration curve, melt curve and amplification blot for each target is illustrated. [file 1297-9716-44-13-S1.pdf]

Additional file 1: Optimisation and validation of qPCR assays for target gene-specific primers in the pig.

Information about Intron-spanning primers.

Primers or products spanning Exon-Exon junctions are indicated in the table including length of intron in base pairs (bp).

Target	Exon junctions in	Intron size (bp)
**NKp46**	product / reverse primer	2,217 / 123
**NKp30**	–	–
**NKG2D**	–	–
**CXCR3**	reverse primer	1,752

Optimised protocol for the amplification of target sequences by qPCR.

Target	Annealing temp (°C)/time (sec)	Extension temp(°C)/time (sec)	ΔCt (RT+ to RT-)	slope	Correlation Coefficient (Pearson) R^2	Verified dynamic range
**NKp46**	62/20	72/30	>8	-3.400	0.999	10^6
**NKp30**	62/20	72/30	>5.5	-3.162	0.998	10^6
**NKG2D**	62/20	72/30	>20	-3.170	0.997	10^6
**CXCR3**	66/30	72/30	>7	-3.335	1.000	10^6

NKp46: 1:2 serial dilution of cDNA pool

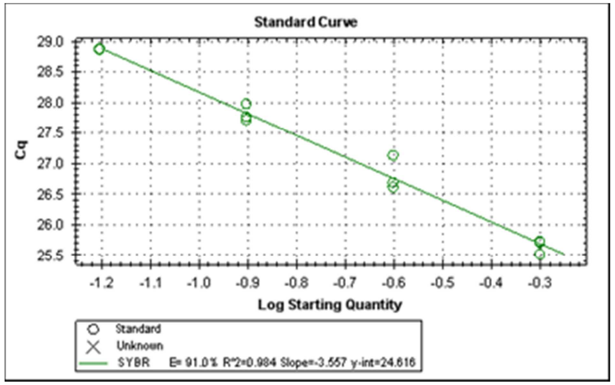

calibration curve

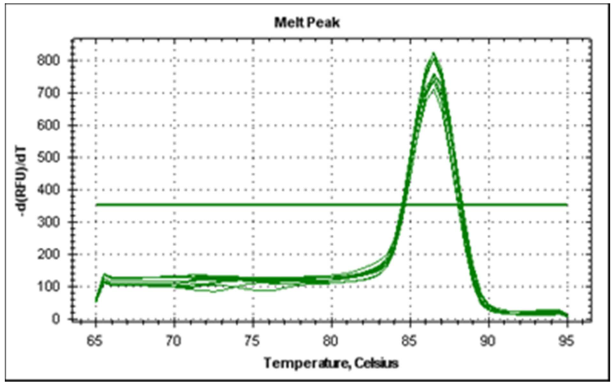

melt curve

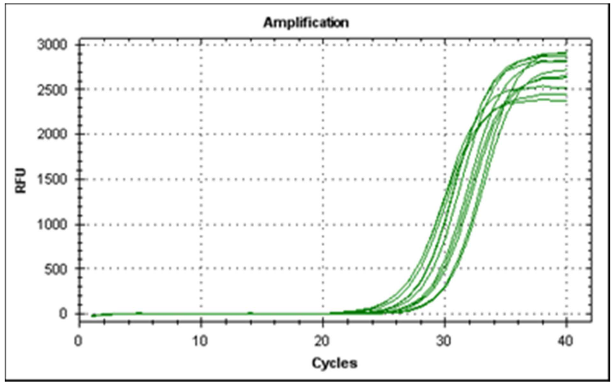

amplification plot

NKp30: 1:10 serial dilution of PCR product

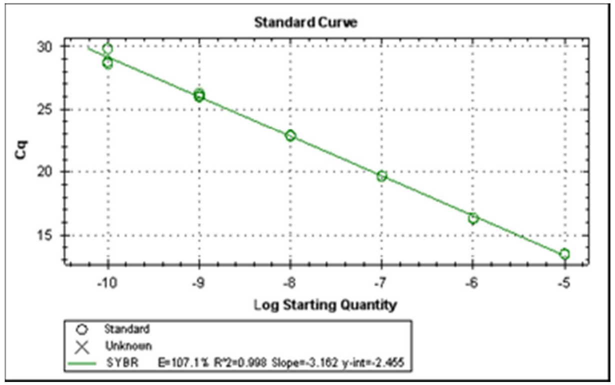

calibration curve

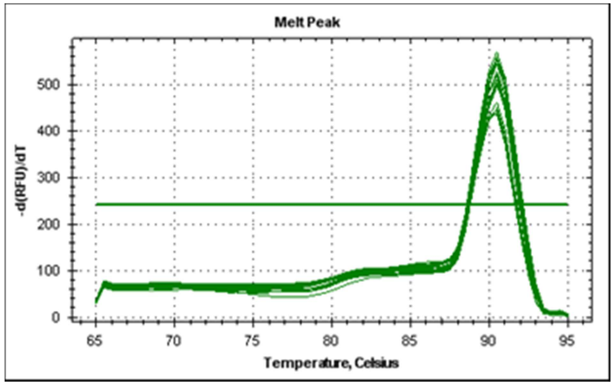

melt curve

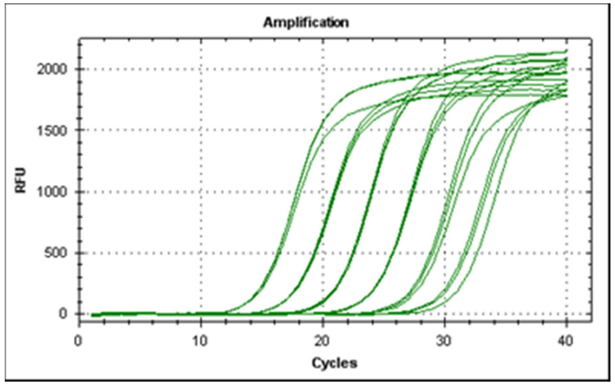

amplification plot

NKG2D: 1:2 serial dilution of cDNA pool

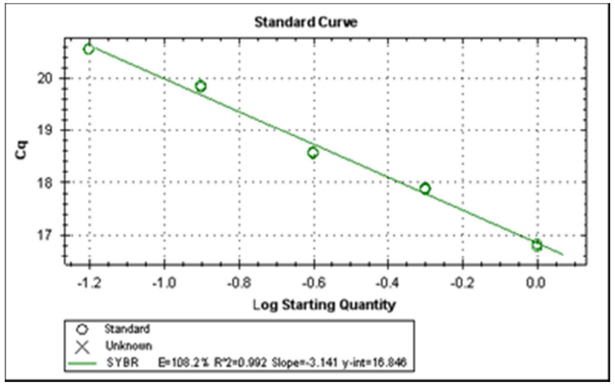

calibration curve

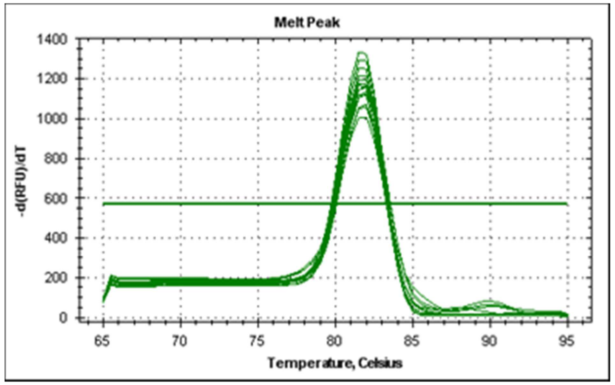

melt curve

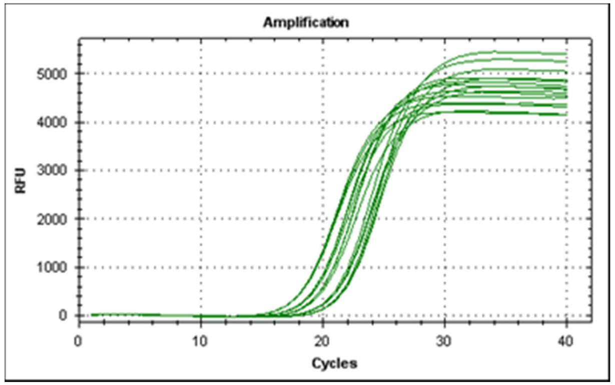

amplification plot

CXCR3: 1:2 serial dilution of cDNA pool

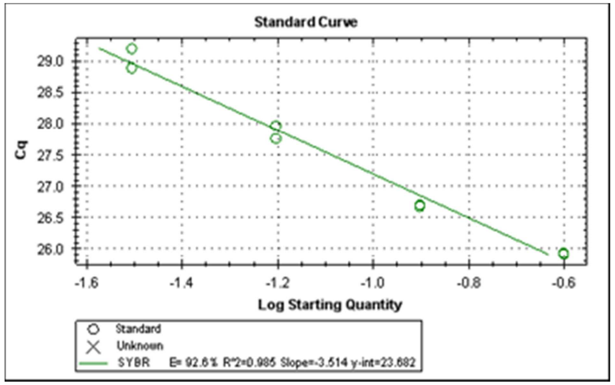

calibration curve

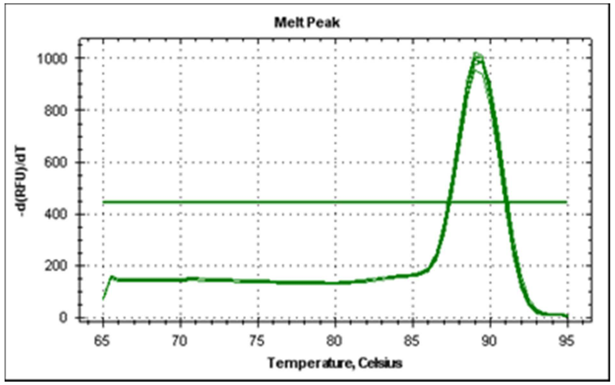

melt curve

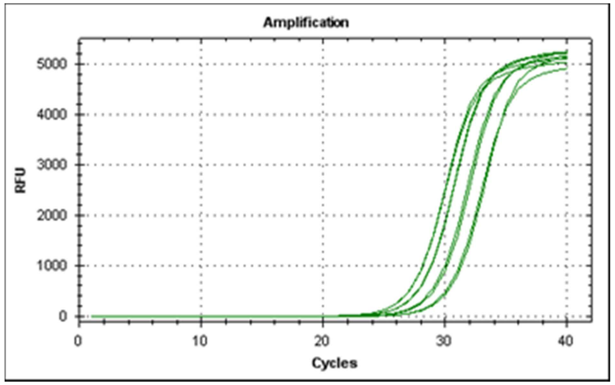

amplification plot
