# Supplementary figures and images for: Porcine CD8αdim/-NKp46high NK cells are in a highly activated state
Source: Vet Res. 2013 Mar 1;44(1):13. doi: 10.1186/1297-9716-44-13 (PMC3599810; doi:10.1186/1297-9716-44-13)

Additional file 2: NK-cell subset gating hierarchy

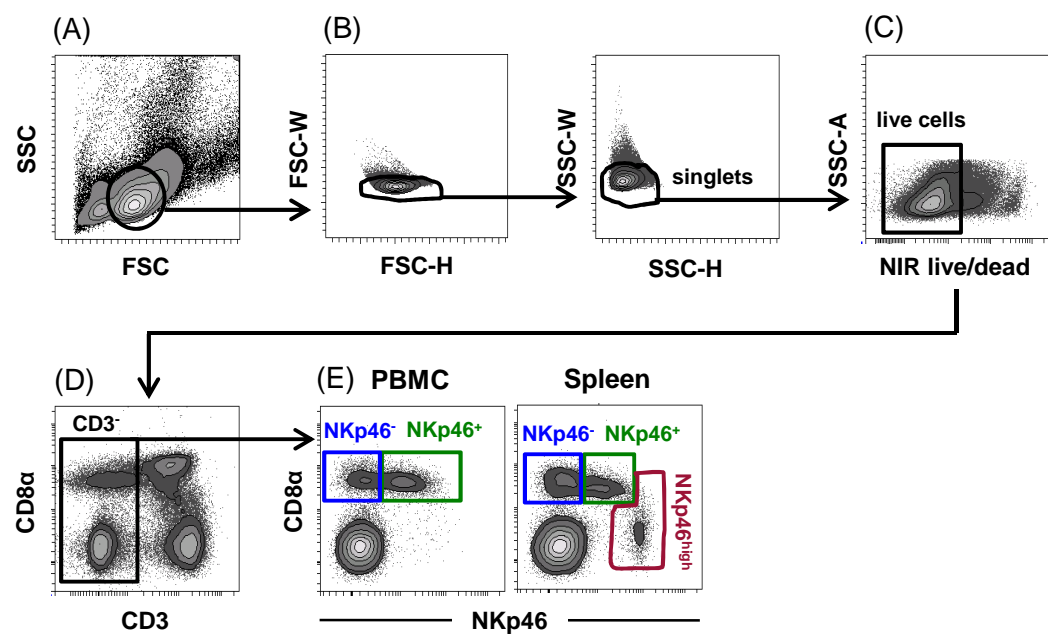

Supplement: Additional file 2 — Gating hierarchy used for FCM analysis of NKp46-defined NK-cell subsets of porcine PBMC and splenocytes. (A) Lymphocytes were gated according to their light scatter properties. (B) To exclude potential doublet cells, a FSC-H/FSC-W gate followed by a SSC-H/SSC-W gate was used. (C) For Live/Dead discrimination, Near-IR stain was used. For further analysis only live cells (Near-IR negative) were included. (D) To exclude T cells, lymphocytes were further gated on CD3- cells. (E) For the identification of different NK subsets CD8α and NKp46 expression was analysed. For PBMC CD3-CD8α+ cells were divided into NKp46- and NKp46+ NK cells. In spleen a third subset could be defined according to its CD8αdim/- and NKp46high phenotype. [file 1297-9716-44-13-S2.pdf]
